# Learning curve of robotic-assisted transabdominal preperitoneal inguinal hernia repair (r-TAPP): a scoping review of CUSUM-based studies

**DOI:** 10.1007/s11701-026-03482-7

**Published:** 2026-05-26

**Authors:** Francesco Brucchi, Rita Stanco, Sofia Esposito, Micaela Piccoli, Giampaolo Formisano, Paolo Pietro Bianchi, Gianlorenzo Dionigi

**Affiliations:** 1 Division of Surgery, Istituto Auxologico Italiano, IRCCS, Via Mercalli 30, Milan, Italy; 2 Department of Pathophysiology and Transplantation, University of Milan, Milan, Italy; 3 University of Pavia, Pavia, Italy; 4 Department of General, Emergency Surgery and New Technologies, Baggiovara General Hospital, AOU Modena, Modena, Italy; 5 Department of Surgery, Dipartimento di Scienze della Salute, ASST Santi Paolo e Carlo, University of Milan, Milan, Italy

**Keywords:** Robotic surgery, Inguinal hernia, TAPP, Learning curve, CUSUM, Scoping review

## Abstract

Robotic-assisted transabdominal preperitoneal repair (r-TAPP) is increasingly adopted for inguinal hernia repair, but the number of procedures required to achieve stable operative performance remains unclear. This scoping review synthesised CUSUM-based learning curve evidence to characterise reported operative-time stabilisation thresholds and their methodological determinants. PubMed, Embase, Scopus, Cochrane Library, and Google Scholar were searched for studies published between January 2000 and December 2025 reporting CUSUM-based learning curve analyses of r-TAPP or SP-TAPP using Da Vinci systems. The review followed PRISMA-ScR guidance and was registered with the Open Science Framework. The primary outcome was the CUSUM inflection point, summarised descriptively using median, sample-size-weighted mean, range, and non-parametric bootstrap 95% confidence intervals. Operative time trends were synthesised narratively without pooling. Seven studies, including eight independent learning curves and 1,361 patients, were included. CUSUM inflection points ranged from 12 to 138 procedures (median 32, 95% CI 13–43; weighted mean 65, 95% CI 22–108). Sensitivity analysis excluding the single series using a composite efficiency-plus-safety CUSUM yielded a median of 29 procedures (95% CI 13–35) and weighted mean of 28 (95% CI 19–36). Both estimates are valid within their methodological contexts. Operative time decreased after stabilisation in all studies by approximately 7–24 min. Complications were predominantly minor; intraoperative events were rare, and reported conversions were absent. Safety comparisons between phases were underpowered. Available evidence is compatible with r-TAPP operative-time stabilisation after approximately 25–35 procedures in structured training settings. This estimate is hypothesis-generating, not a credentialing benchmark, given heterogeneous CUSUM definitions and very low-certainty evidence.

**Supplementary Information:**

The online version contains supplementary material available at 10.1007/s11701-026-03482-7.

## Introduction

Inguinal hernia repair is one of the most commonly performed surgical procedures worldwide, with an estimated 20 million operations carried out annually [1–3]. Over the past three decades, the field has shifted progressively from open anterior repair toward minimally invasive approaches [2], aiming to reduce postoperative morbidity, accelerate recovery, and improve patient-reported outcomes [4].

Minimally invasive inguinal hernia repair — whether laparoscopic or robotic — is now widely accepted for bilateral hernias and increasingly adopted for unilateral defects, particularly in working-age patients [4]. Among available techniques, transabdominal preperitoneal repair (TAPP) offers excellent visualisation of the inguinal anatomy and allows bilateral repair through the same access [3, 5]. The robotic platform has expanded the application of TAPP further: robotic-assisted TAPP (r-TAPP) provides improved instrument articulation, three-dimensional visualisation, and better ergonomics, potentially facilitating preperitoneal dissection and mesh placement [3].

However, adopting r-TAPP requires surgeons to acquire a specific set of technical and cognitive skills. This process is commonly described as a learning curve — a period during which performance improves with accumulated experience [6–8]. Understanding this trajectory matters: it informs the design of safe training pathways, helps benchmark institutional performance, and may help minimise the risk of compromising outcomes during early experience. Surgeon-level determinants — prior laparoscopic volume, simulation hours, structured proctorship, and exposure to other robotic procedures — are all known to influence the learning trajectory, although their relative contributions are rarely quantified.

Cumulative sum (CUSUM) analysis is one of the most widely used statistical tools for evaluating surgical learning curves [9, 10]. By tracking sequential deviations from a predefined performance target, CUSUM identifies inflection points marking the transition from skill acquisition to stable performance on the selected outcome [10]. A crucial methodological point is that the CUSUM inflection point is a function of the variable on which the CUSUM is built. When CUSUM is applied to operative time — as in the majority of published r-TAPP studies — the inflection point identifies the stabilisation of operative efficiency, not clinical proficiency in a broader sense. When CUSUM is risk-adjusted to incorporate complications and surgical site events (RA-CUSUM), it identifies a different, more demanding endpoint — the simultaneous stabilisation of efficiency and perioperative safety. Throughout this review we therefore use the term “operative-time stabilisation point” for the standard CUSUM inflection on operative time, and reserve “proficiency” for the broader composite of efficiency, safety, and longer-term outcomes.

For laparoscopic TAPP, the learning curve has been estimated at 20–50 procedures in most series [11–13]. Whether these figures apply to the robotic platform — which poses different ergonomic and visuospatial demands — is unclear. As robotic systems beyond Da Vinci (Hugo RAS, Versius, Senhance) enter the market, procedure-specific learning data and training pathways become all the more relevant for training standardisation [6, 14].

Several single-centre studies have used CUSUM-based methods to investigate the r-TAPP learning curve, but the evidence remains fragmented: surgeon backgrounds, institutional volumes, case-mix, and outcome definitions vary considerably, and formal inferential synthesis is precluded because individual-level variance estimates for CUSUM inflection points are rarely reported.

The aim of this scoping review was therefore to map the CUSUM-based evidence on the r-TAPP learning curve, to describe the range of reported operative-time stabilisation thresholds, and to identify the contextual factors that modulate them.

## Materials and methods

### Design and registration

This review was conducted in accordance with the Preferred Reporting Items for Systematic Reviews and Meta-Analyses extension for Scoping Reviews (PRISMA-ScR) [15]. A scoping review framework was chosen because the available evidence — retrospective single-centre series reporting CUSUM inflection points without individual-level variance estimates — is not suitable for formal inferential meta-analysis. The PRISMA-ScR framework guided the review at three operational levels: (i) study selection, by emphasising broad inclusion of any CUSUM-based learning-curve evidence rather than restricting to a single comparator; (ii) synthesis, by privileging descriptive mapping over inferential pooling; and (iii) interpretation, by framing findings as a description of the existing knowledge base rather than as causal estimates of effect. The protocol was registered with the Open Science Framework (OSF; registration DOI to be inserted at proof stage).

### Search strategy

We searched PubMed, Embase, Scopus, and the Cochrane Library for peer-reviewed literature published between January 1, 2000, and December 31, 2025. To address the possibility of relevant unpublished or non-indexed work, we additionally screened the first 200 results of a Google Scholar query and hand-searched the bibliographies of retrieved articles and relevant reviews. Conference proceedings and abstracts without an associated full publication were not included, on the basis that they typically lack the methodological detail required for CUSUM extraction; this is acknowledged as a possible source of selection bias. The full search strategies for each database are detailed in Supplementary Fig. 1s.

### Study selection

Two investigators (FB, RS) independently screened records using Rayyan [16], first by title and abstract, then by full-text review. Disagreements were resolved by a third reviewer (GD). Inclusion criteria were: (1) English-language articles; (2) adult patients (≥ 18 years) undergoing inguinal hernia repair; (3) r-TAPP or SP-TAPP performed with Da Vinci robotic systems; (4) learning curve evaluation using CUSUM or CUSUM-derived methods; (5) reporting of at least one learning-curve outcome (inflection point, operative time trends, or phase transitions).

No geographic restrictions were applied. Conference abstracts, narrative reviews, and duplicate reports were excluded. Studies using techniques other than TAPP (e.g., totally extraperitoneal repair) were excluded because the different surgical approach and robotic interface represent independent determinants of the learning trajectory. Paediatric series were not eligible; the review consequently does not address congenital versus acquired aetiology or peritoneal-vaginal duct persistence, which are relevant only in paediatric inguinal hernia.

### Data extraction

Two authors independently extracted: first author, year, country, study period, design, number of surgeons and procedures, surgeon background and prior experience (laparoscopic TAPP volume, prior robotic exposure, simulation training, proctorship), and institutional context. Patient and case characteristics (age, sex, BMI, ASA score, hernia type and laterality, complex hernia features) were recorded when available. Learning curve data included the CUSUM method used (standard CUSUM, risk-adjusted CUSUM, or both), the outcome variable driving the CUSUM curve, the inflection point or phase transitions, operative time trends, complications (Clavien–Dindo when available), specific complication types (seroma, haematoma, surgical site infection, urinary retention, visceral injury, mesh-related events), conversion rates, length of stay, and any reported long-term outcomes (recurrence, chronic pain, return to activity).

### Risk of bias and certainty of evidence

Methodological quality was assessed with the Newcastle–Ottawa Scale (NOS) [17]. The certainty of evidence was rated using the GRADE framework [18]. Two authors performed these assessments independently (FB, RS); disagreements were resolved by discussion.

### Outcome definitions

#### Operative-time stabilisation point

The primary variable of interest was the CUSUM inflection point on operative time, interpreted as the point at which operative efficiency stabilises. Across individual studies this is variably labelled “proficiency”, “learning phase completion”, or “performance stabilisation”; these terms are not strictly interchangeable, and we treat them throughout as a common descriptive marker of operative-time stabilisation rather than as a unified proficiency construct. Where a study reported more than one inflection point (e.g., successive phases), the first one was used as the primary stabilisation threshold; additional inflections were recorded separately as markers of later optimisation. Where a study built its CUSUM on a composite endpoint incorporating safety outcomes (risk-adjusted CUSUM), this is flagged explicitly because such constructions identify a different and more demanding endpoint.

#### Secondary variables

Secondary variables included operative time trends across phases, complication rates and types, intraoperative events, conversion to open or laparoscopic surgery, length of stay, and the number of learning phases reported. Subgroup mapping was planned by surgeon experience, case complexity, and hernia laterality, as data permitted.

### Synthesis approach

Given the heterogeneity in CUSUM parameterisation, the unavailability of individual-level variance estimates for inflection points, and the inconsistent definitions of operative time across studies, no inferential meta-analytic pooling was attempted. We acknowledge that descriptive aggregation — even when accompanied by bootstrap confidence intervals — simplifies methodological differences between studies. The summary statistics presented below should therefore be read as a structured description of the existing evidence, not as estimates of a common underlying parameter. Specifically:

- Inflection points were summarised descriptively using the median, sample-size-weighted mean, and range. Non-parametric bootstrap 95% confidence intervals (10,000 resamples with replacement) were computed around the median and the weighted mean to convey the precision of these point estimates without assuming a parametric distribution.
- A pre-specified sensitivity analysis excluded the single series that built its CUSUM on a composite efficiency-plus-safety endpoint (Kudsi et al. [9], who reported both standard CUSUM on operative time and a risk-adjusted CUSUM incorporating complications). This series is reported in the primary analysis as a valid data point, and excluded in the sensitivity analysis to estimate the inflection point under a homogeneous methodological framework (operative-time-only CUSUM). Both estimates are presented as valid within their respective methodological contexts; neither is privileged.
- Operative time trends were visualised in a descriptive forest plot showing study-specific mean differences between pre- and post-stabilisation phases. No pooled estimate is presented because between-study heterogeneity in operative time definitions (skin-to-skin, console time, corrected time) and in case-mix precludes a meaningful summary effect; this limitation is reinforced graphically by the absence of a summary diamond.
- Where medians and interquartile ranges were reported, they were converted to means and standard deviations using the method of Wan et al. [19] for descriptive consistency only.

Complications and other secondary outcomes were described narratively, with explicit recognition that the included studies are individually and collectively underpowered to detect meaningful differences in safety endpoints between learning-curve phases. All analyses were performed in Python 3 using NumPy and statsmodels; figures were generated with matplotlib.

## Results

### Study selection

**Fig. 1:**
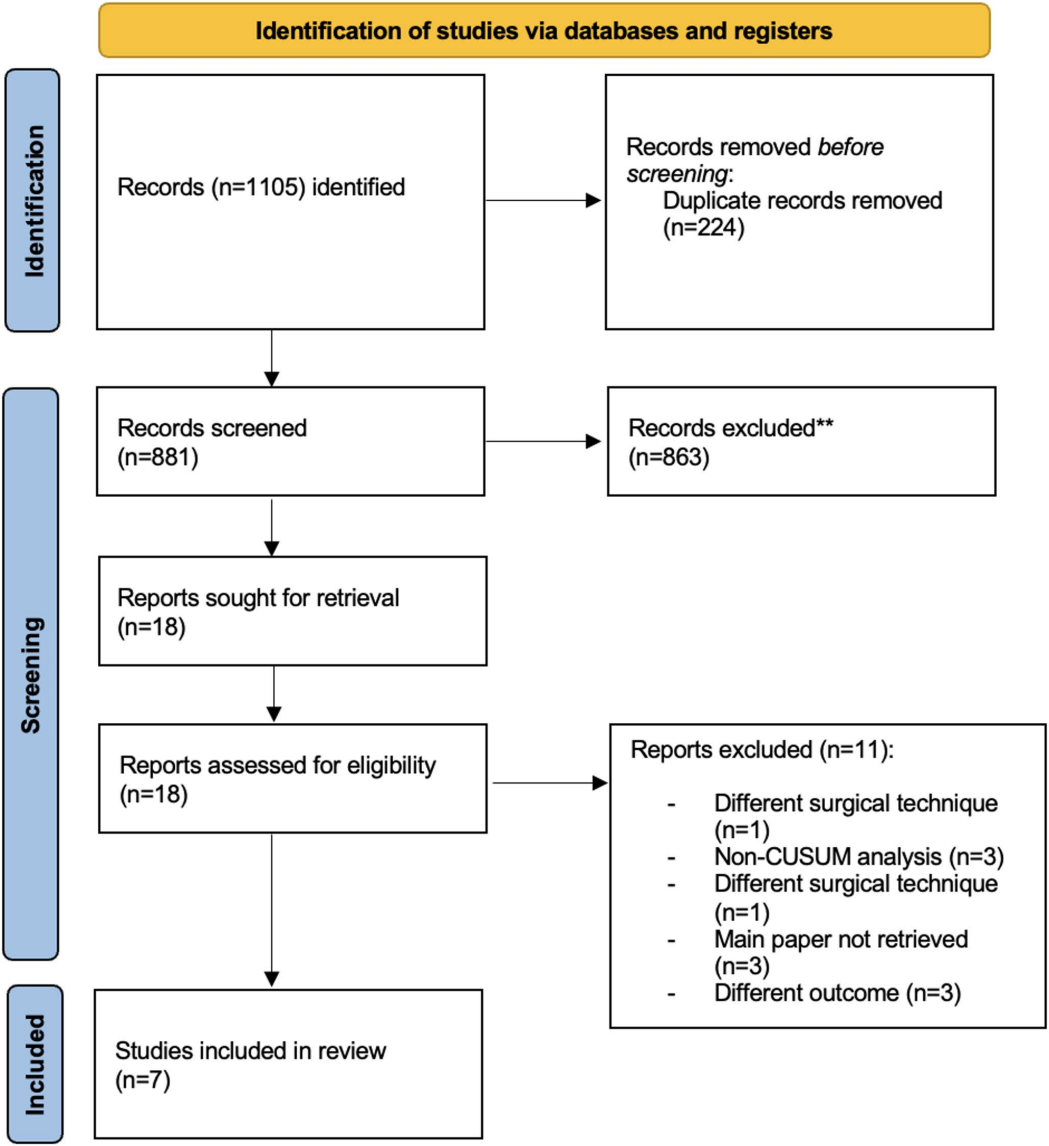
PRISMA-ScR flow diagram of study selection

The search retrieved 1105 records. After removing 224 duplicates, we screened 881 titles and abstracts and assessed 18 full texts. Seven studies met the inclusion criteria (Fig. 1) [9, 11–13, 20–22].

### Study characteristics

All seven studies were retrospective series published between 2020 and 2025, from five countries, enrolling a total of 1,361 patients (range 50–462 per series; Table 1) [9, 11–13, 20–22]. The enrolment period spanned February 2013 to September 2024. All procedures used Da Vinci platforms; one series [12] employed the Single Port system (SP-TAPP). Four series reported single-surgeon curves [9, 11, 12, 20], two included two surgeons [21, 22], and one three [13]. Solaini et al. (2023) [21] is the only study specifically comparing expert and trainee learning curves within the same cohort. Five studies described structured pre-clinical training (simulation, dry/wet lab, cadaver/animal sessions, or proctored initial cases) [9, 12, 13, 21, 22].

**Table 1:** Characteristics of included studies

Author	Year	Country	Enrolment	Platform	Technique	Total pts	*N* surgeons	Pts per phase	Prior surgeon experience	CUSUM outcome
Celotto et al.	2025	Italy/USA	Jul 2019 – Apr 2024	Da Vinci SP	SP-TAPP	122	1	P1: 13; P2: 100; P3: 9	Exp. in lap TAPP, robotic multiport, DV single-site; SP-specific training; proctored first cases	OT only
Solaini et al.	2025	Italy	Jan 2020 – Sep 2024	Da Vinci Si/Xi	TAPP	462 (245 rob.)	2	Learn: 58; Post: 187	MIS colorectal; one expert (~ 80/yr) + one junior (simulator, dry/wet lab, > 150 assisted)	OT + mixed-effects
Choi et al.	2023	Korea	Nov 2020 – Jun 2022	Da Vinci Xi	TAPP	100 (50 rob.)	1	Learn: 12; Post: 38	Extensive laparoscopic TAPP (> 100 TEP+TAPP)	Console time
Solaini et al.	2023	Italy	Jan 2020 – Dec 2022	Da Vinci Si/Xi	TAPP	124+	2 (A: expert; B: trainee)	A: 35/26; B: 20/43	No MIS TAPP; A: expert robotic colorectal (~ 80/yr); B: basic robotic + ~ 20 supervised	OT (+ per-step)
Kudsi et al.	2022	USA	Feb 2013 – Jan 2018	Da Vinci Si (86%)/Xi	TAPP	371	1	Learn: 138; Post: 233	MIS-trained; no robotic fellowship; ~150 lap IHR + ~ 40 robotic prior. Early era.	OT + RA-CUSUM
Aghayeva et al.	2020	Turkey	Apr 2016 – Oct 2019	Da Vinci Xi	TAPP	50	1	Learn: 35; Post: 15	Completed LC for lap inguinal; > 400 robotic abd. within study period	OT only
Proietti et al.	2021	Switzerland	Dec 2017 – Dec 2019	Da Vinci Si/Xi	TAPP	132	3	Learn: 97; Post: 35	All 3 expert lap TAPP (> 150 each); no prior robotic; robotic training + sim	Corrected OT

OT, operative time; MIS, minimally invasive surgery; SP, single port; RA-CUSUM, risk-adjusted CUSUM; IHR, inguinal hernia repair

### Patient and hernia characteristics

The population was predominantly male, with mean ages of 48–67 years and BMIs of 24.5–27.7 kg/m², indicating a broadly homogeneous, non-obese cohort (Table 2). ASA I–II predominated in the four studies reporting this variable [9, 12, 20, 21]. Hernia classification systems differed across series (direct/indirect/femoral [9, 20], EHS [21, 22], Nyhus [12]), which limits cross-study comparison of case complexity (Table 3). Complex hernias — variably defined as recurrent, incarcerated, scrotal, or post-prostatectomy — were present in 6–21% of cases across five series, and were not systematically excluded during the learning phase in any study. Bilateral hernia rates ranged from 0% to 49%, a substantial source of clinical heterogeneity. None of the included studies reported the proportion of inguinal hernia repairs performed robotically at the institutional level, the criteria used to allocate cases to a robotic versus laparoscopic approach, or the anaesthetic protocol used (e.g., opioid-free pathways).

**Table 2:** Patient characteristics

Author	Age pre-learning	Age post-learning	Sex (M/F) pre	Sex (M/F) post	BMI pre	BMI post	ASA pre	ASA post
Celotto et al.	P1: 54 (38–58)*; P2: 58 (45–67)*	P3: 51 (46–68)*	P1: all M; P2: 89/11	P3: all M	P1: 24.9; P2: 25.8	P3: 29.3	P1: I:5/II:6/III:2; P2: I:16/II:56/III:27/IV:1	P3: II:5/III:4
Solaini et al. 2025^§^	61 (56–73)*	67 (55–75)*	54/4	176/11	25.9	24.5	NA	NA
Choi et al.	54.4 ± 14	54.4 ± 14	All M	All M	24.8 ± 3	24.8 ± 3	I:3/II:43/III:4	I:3/II:43/III:4
Solaini et al. 2023	NR	A: 68 (48–78)*; B: 60 (49–74)*	NA	A: 24/2; B: all M	NA	A: 24.9; B: 24.7	NA	A: ≥II 38.5%; B: ≥II 27.9%
Kudsi et al.	59.3 ± 15.6	59.6 ± 16.2	118/20	217/16	27.7 ± 4.2	27.4 ± 5	I:15%/II:60%/III:25%	I:9%/II:51%/III:40%
Aghayeva et al.	52.8 ± 16.3	48.3 ± 18	33/2	All M	25 ± 3.2	25 ± 2.9	NA	NA
Proietti et al.	60.1 ± 14.1	60.1 ± 14.1	All M	All M	26.3 ± 3.0	25.4 ± 3.0	NA	NA

*Median (range). ^§^Pooled data for two surgeons. Ages in years; BMI in kg/m²

**Table 3:** Hernia characteristics

Author	Hernia type pre	Hernia type post	Side pre	Side post	Complex pre	Complex post
Celotto et al.	Nyhus P1: 1–4; P2: mostly type 2	Nyhus P3: 1–3a	All unilateral	All unilateral	NA	NA
Solaini et al. 2025	EHS L1–3, M1–3	EHS L1–3, M1–3, Femoral 3	Right 48.3%	Right 49.7%	Recurrent 6.9%	Recurrent 8.5%
Choi et al.	Indirect 86%; Direct 8%; Mixed 4%; Femoral 2%	Indirect 86%; Direct 8%; Mixed 4%; Femoral 2%	R 58%; L 36%; Bilat 6%	R 58%; L 36%; Bilat 6%	Recurrent 6%; Incarcerated 10%; Post-prostatectomy 4%	Same as pre
Solaini et al. 2023	NA	A EHS I/II/III; B EHS I/II/III	NA	A: Right 53.8%; B: Right 48.8%	NA	A: Recurrent 15.4%; B: 9.3%
Kudsi et al.	Direct 29.7%; Indirect 88.4%; Femoral 3.6%	Direct 38.2%; Indirect 73.8%; Femoral 1.3%	Unilat 71%; Bilat 29%	Unilat 70.8%; Bilat 29.2%	18.8%†	21%^†^
Aghayeva et al.	NA	NA	Bilat 48.6%	Bilat 33.3%	NA	NA
Proietti et al.	NA	NA	NR	NA	Recurrent 6.2%	Recurrent 14.3%

^†^Complex hernias: prior prostatectomy, incarcerated, scrotal, recurrent, or previous anterior/posterior repair

### Risk of bias and GRADE assessment

All seven studies scored 8/9 on the NOS (Supplementary Table 1s). The main limitations were those intrinsic to retrospective learning-curve research: no randomisation, potential selection bias, and limited control over confounders such as prior experience and institutional context. No randomised trials were identified.

GRADE certainty was very low for all outcomes (Supplementary Table 2s). For the operative-time stabilisation point, this was driven by heterogeneity in inflection points and inconsistent definitions of the CUSUM threshold. For operative time trends, substantial clinical and methodological heterogeneity further reduced certainty. For complications and other secondary outcomes, sparse events, incomplete phase-specific reporting, and individually underpowered comparisons were additional limiting factors.

### CUSUM findings

#### Operative-time stabilisation point

All seven studies applied CUSUM analysis to operative time [9, 11–13, 20–22]. Eight data points were available (Solaini et al. 2023 contributed two independent surgeon-level curves). The inflection point ranged from 12 [20] to 138 procedures [9], with a median of 32 (bootstrap 95% CI 13–43) and a sample-size-weighted mean of 65 (95% CI 22–108) (Table 4; Fig. 3). The pre-specified sensitivity analysis excluding the Kudsi et al. series — which built its CUSUM on a composite efficiency-plus-safety endpoint via risk-adjusted CUSUM — yielded a median of 29 (95% CI 13–35) and a weighted mean of 28 (95% CI 19–36). The two analyses correspond to different methodological framings (operative-time only versus operative time plus safety) and should be read as such; neither supersedes the other.

**Table 4:** CUSUM analysis results

Author	Inflection point(s)	OT pre	OT post	OT definition	CUSUM outcome	Notes
Celotto et al.	13th; 113th	P1: 81 (78–93)	P3: 61 (48–65)	Skin-to-skin	OT only	SP-TAPP; 2nd IP = later optimisation, not primary stabilisation
Solaini et al. 2025	29th (each surgeon)ᵃ	83 (73–104)	59 (46–69)	Skin-to-skin	OT only	Pooled 2 surgeons; also mixed-effects model
Choi et al.	12th	35.5 ± 17.0ᵇ	28.6 ± 9.4ᵇ	Console timeᵇ	Console time	Shortest learning phase; ᵇ console time not skin-to-skin
Solaini et al. 2023	A: 35th; B: 20th	A: 67 (60–75); B: 90 (80–105)	A: 52 (45–65); B: 65 (53–75)	Skin-to-skin	OT + per-step	Expert (A) vs. trainee (B); structured mentorship
Kudsi et al.	138th	58 (47–80)	45 (36–61)	Skin-to-skin	OT + RA-CUSUM	Longest learning phase; single high-volume surgeon; early robotic era
Aghayeva et al.	35th	120 ± 41.3	95.6 ± 30.8	Skin-to-skin	OT only	—
Proietti et al.	43rdᵃ	71.1 ± 22.0ᶜ	60.8 ± 13.5ᶜ	Corrected OTᶜ	Corrected OT	3 surgeons pooled; ᶜ bilateral ÷ 2, minus 25.5 min for unilateral

ᵃBilateral hernias counted as two procedures

ᵇConsole time, not skin-to-skin; systematically shorter and lower variance

ᶜCorrected OT = bilateral OT ÷ 2, minus 25.5 min for unilateral hernias. OT values in minutes, expressed as median (IQR) or mean ± SD

Among single-surgeon series, inflection points were 12 [20], 35 [11], 43 ([13], 3 surgeons pooled), and 138 [9]. Solaini et al. (2025) [22] found a common threshold at 29 procedures for both surgeons. In the Solaini et al. (2023) [21] series, the trainee (Surgeon B) reached stabilisation at 20 cases — earlier than the expert (Surgeon A) at 35 — a finding the authors attributed to structured mentorship; this observation derives from a single study and cannot be generalised. Celotto et al. [12] identified two inflection points (13th and 113th case); the first was used as the primary stabilisation threshold, and the second is discussed separately as a marker of later optimisation.

#### Operative time trends

**Fig. 2:**
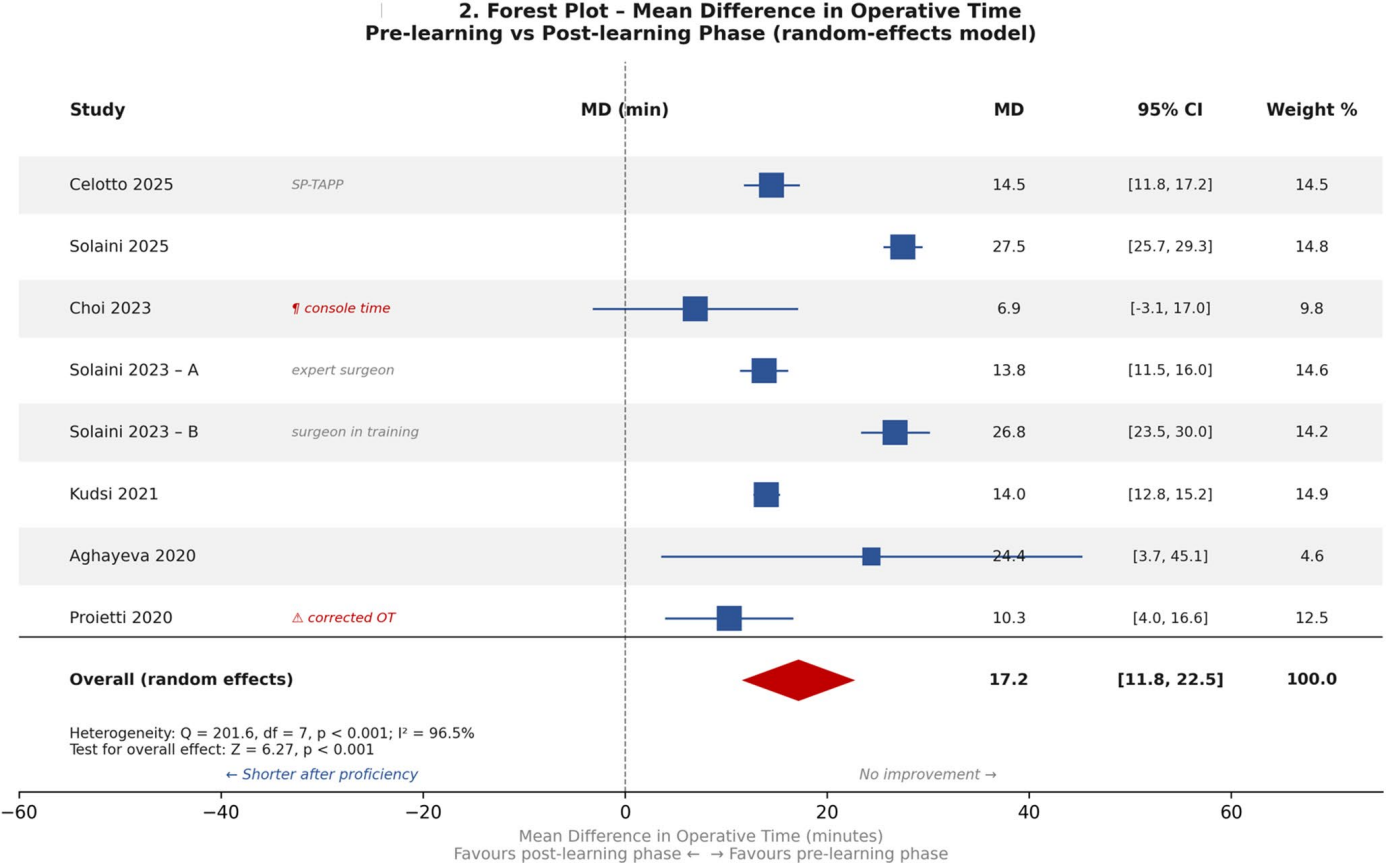
Forest plot of study-specific mean differences in operative time between pre- and post-stabilisation phases. The figure is presented for visual comparison only; no pooled estimate (summary diamond) is shown, because heterogeneity in operative time definitions (skin-to-skin, console time, corrected time) and case-mix precludes a meaningful summary effect. Readers should not interpret the alignment of individual study point estimates as evidence of a common underlying effect size

Operative time decreased consistently across all series (Table 4; Fig. 2). The within-study reduction between pre- and post-stabilisation phases ranged from approximately 7 min (Choi et al., console time [20]) to 24 min (Solaini et al. 2025 [22]). Five studies reported skin-to-skin time [9, 11, 12, 21, 22]; Choi et al. used console time, systematically shorter and with lower variance [20]; Proietti et al. [13] used a normalised “corrected OT” to account for bilateral procedures. Kudsi et al. [9] reported both metrics, providing the only direct intra-study comparison: console time fell from a median of 45 to 34 min (*p* < 0.001), while skin-to-skin time fell from 58 to 45 min (*p* < 0.001), with the console-to-skin-to-skin ratio remaining around 0.76 in both phases. This suggests that, when applied to operative-time CUSUM, console time may stabilise on a similar trajectory but at a systematically shorter absolute scale than skin-to-skin time, biasing inflection-point comparisons across studies that use different metrics. Because of these heterogeneous definitions and the wide variation in case-mix (bilateral hernia rates 0–49%; complex hernia rates 6–21%), we did not compute a pooled effect size; the forest plot in Fig. 2 is therefore presented for visual comparison only, without a summary diamond.

#### Complications and intraoperative events

**Fig. 3:**
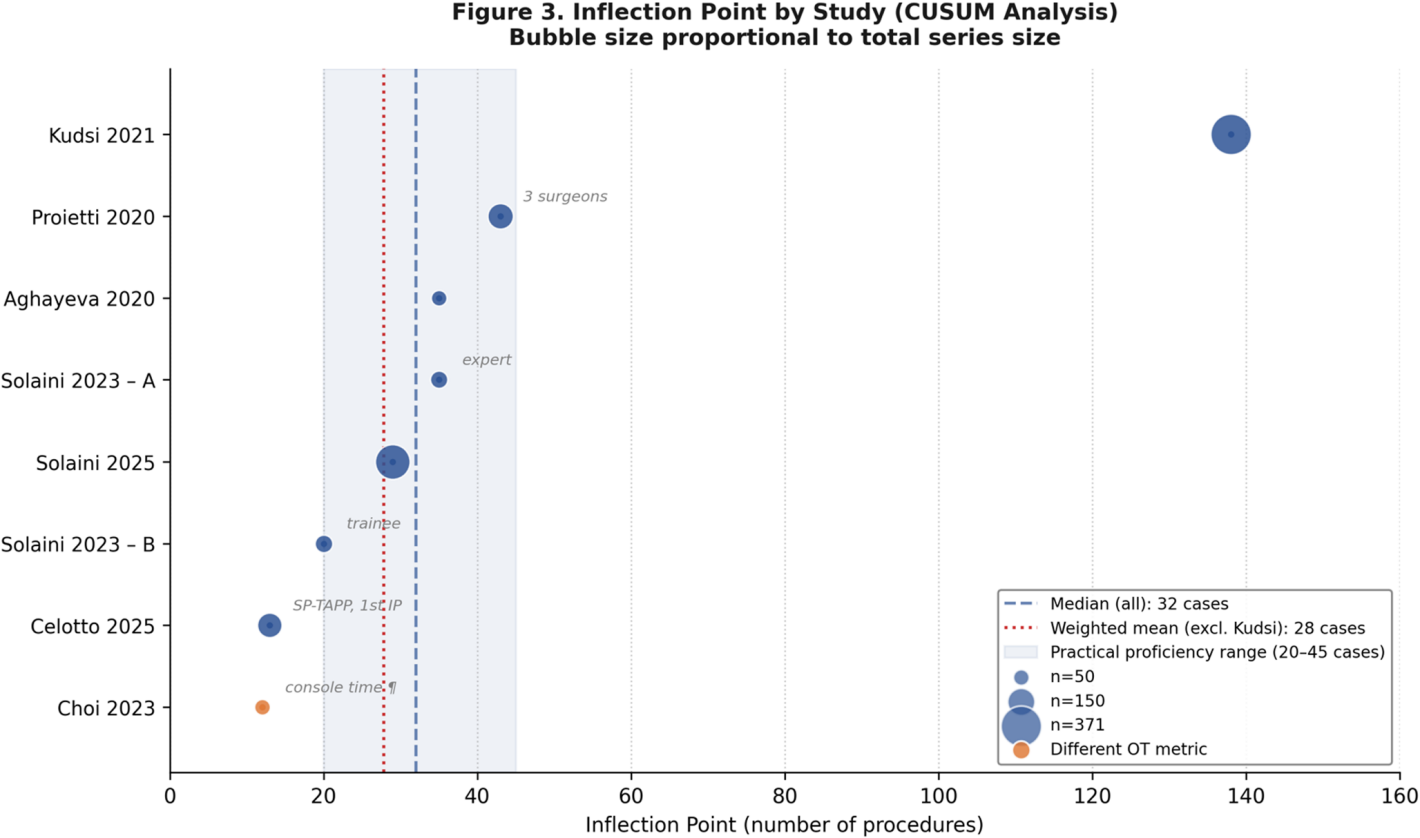
CUSUM operative-time stabilisation points by study. Bubble size is proportional to total series size. Dashed vertical lines indicate the median across all eight curves (32 procedures) and the median under the homogeneous operative-time-only sensitivity analysis (29 procedures). The two values represent different methodological framings rather than alternative point estimates of a single quantity

Postoperative complications were predominantly minor across all series (Table 5). The most commonly reported events were seromas (range 0–9%), haematomas (range 1–6%), and urinary retention (range 0–10%); surgical site infections were rare (0–3%). Intraoperative events were rare and reported in three series only: Proietti et al. [13] reported a single testicular vessel injury during reduction of an inguino-scrotal hernia, requiring orchiectomy (during the learning phase); Kudsi et al. [9] reported one serosal tear (early phase) and one stomach injury during Veress needle insertion (late phase), both managed with simple suture; Choi et al. [20] reported one bladder injury related to tacker slippage, managed conservatively. Aghayeva et al. [11] explicitly reported no intraoperative complications (including no vascular, cord, bladder, or bowel injury), and Celotto et al. [12] reported no intraoperative complications across 122 SP-TAPP cases. Major postoperative events (Clavien–Dindo ≥ III) were uncommon: Proietti et al. [13] reported three Clavien–Dindo III–IV events during the learning phase (one drained seroma, one drained haematoma, and one mesh infection requiring surgical removal of the prosthesis) and none after stabilisation, whereas Kudsi et al. [9] reported four such events across the early phase and two in the late phase, with significantly lower rates of surgical site events (8.8% vs. 2.2%, *p* = 0.008) and surgical site occurrences requiring procedural intervention (2.9% vs. 0.4%, *p* = 0.047) after stabilization (Fig. 3).

**Table 5:** Detailed complication profile and intraoperative events

Author	Phase	Seroma	Haematoma	SSI	Urinary retention	Visceral/vascular injury	Major (Clavien ≥ III)	Conversion
Celotto et al.	Overall (*n* = 122)	NR	NR	NR	NR	0 intraoperative	0	0/122
Solaini et al. 2025	Learning (*n* = 58)	NR	NR	NR	NR	NR	0/58	NR
Solaini et al. 2025	Post (*n* = 187)	NR	NR	NR	NR	NR	2/187 (1.1%)	NR
Choi et al.	Overall (*n* = 50)	0	1 (2%)	0	3 (6%)	1 bladder injury (tacker, post-op)	0	0/50
Solaini et al. 2023	Surgeon A learning (*n* = 35)	NR	NR	NR	NR	NR	0	NR
Solaini et al. 2023	Surgeon B learning (*n* = 20)	NR	NR	NR	NR	NR	0	NR
Kudsi et al.	Early (*n* = 138)	8 (5.9%)	2 (1.5%)	2 (1.5%)	NR	1 serosal tear	4 (2.9%)^†^	0
Kudsi et al.	Late (*n* = 233)	1 (0.4%)	3 (1.3%)	1 (0.4%)	NR	1 stomach (Veress)	2 (0.9%)^†^	0
Aghayeva et al.	Phase I (*n* = 35)	5 (15%)	0	0	0	0	0	0/50
Aghayeva et al.	Phase II (*n* = 15)	1 (6.7%)	0	0	0	0	0	0/50
Proietti et al.	Learning (*n* = 129)	2 (2.1%)	6 (6.2%)	2 (2.1%)	2 (2.1%)	1 testicular vessel injury → orchiectomy	3 (3.1%)^‡^	0/170
Proietti et al.	Post (*n* = 41)	0	1 (2.9%)	1 (2.9%)	2 (5.7%)	0	0	0/170

^†^Kudsi et al. Clavien ≥ III: early phase 3 IIIa + 1 IIIb; late phase 1 IIIb + 1 IVa. ^‡^Proietti et al. learning-phase Clavien III–IV: one drained seroma, one drained haematoma, one mesh infection requiring surgical removal. NR, not reported. The studies are individually and collectively underpowered to detect clinically meaningful differences in safety outcomes between phases; absence of statistical significance should not be read as evidence of equivalence

Among the studies with phase-stratified data, complication rates trended lower after the stabilisation point in five of six series (aggregate crude rates: 14.1% pre vs. 10.0% post), with the difference reaching statistical significance only in the largest series [9]; the pooled and individual analyses are individually underpowered to detect clinically meaningful differences in safety outcomes between phases, and the absence of a statistically significant difference should not be interpreted as evidence of equivalence.

Conversion to open or laparoscopic surgery was explicitly reported in five studies: Celotto et al. (0/122) [12], Choi et al. (0/50) [20], Kudsi et al. (0/371) [9], Proietti et al. (0/170) [13], and Aghayeva et al. (0/50) [11]. No conversions occurred in any study reporting on this outcome (0/763 procedures). Length of stay did not differ between phases in any series; all patients in the Celotto cohort were discharged on the day of surgery [12]. Long-term follow-up was variable: Proietti et al. [13] reported no recurrences at a mean 11.7 months (overall cohort), while Kudsi et al. [9] reported a mean follow-up of 51 months but did not stratify recurrence or chronic pain by learning-curve phase. None of the included studies reported phase-stratified recurrence, chronic pain, or return-to-activity outcomes — the long-term endpoints that ultimately define the quality of an inguinal hernia repair (Table 6).

**Table 6:** CUSUM operative-time stabilisation points — descriptive synthesis with bootstrap 95% CI

Analysis	*N* curves	Median (95% CI)	Weighted mean (95% CI)	Range
Primary (all curves)	8	32 (13–43)	65 (22–108)	12–138
Sensitivity (excluding Kudsi)	7	29 (13–35)	28 (19–36)	12–43

Non-parametric bootstrap with 10,000 resamples with replacement; percentile 95% confidence intervals. Weights for the weighted mean are each curve’s total patient count. The two analyses correspond to different methodological framings: the primary analysis includes all reported inflection points regardless of whether the underlying CUSUM was built on operative time alone or on a composite endpoint, while the sensitivity analysis is restricted to operative-time-only CUSUM curves. Both estimates are valid within their respective contexts; neither is privileged

## Discussion

This is, to our knowledge, the first systematic mapping of CUSUM-based learning curve data for r-TAPP. Across seven studies and eight independent learning curves [9, 11–13, 20–22], the median operative-time stabilisation point was 32 procedures (range 12–138). A pre-specified sensitivity analysis under a homogeneous methodological framework (operative-time-only CUSUM) yielded a median of 29 and a weighted mean of 28; both estimates are valid within their respective contexts and neither should be privileged over the other. Operative time decreased in every included series after the inflection point, typically by 10–25 min — a difference that is clinically meaningful in terms of anaesthetic exposure, theatre throughput, and cost, even though cross-study pooling is not appropriate given the heterogeneity in operative time definitions and case-mix.

The tenfold range in reported thresholds (12–138) is best understood as a function of methodology and context rather than as a single biological signal. At one extreme, Choi et al. [20] reported stabilisation after just 12 procedures. That surgeon, however, had extensive laparoscopic TAPP experience, and the CUSUM was built on console time — a shorter, lower-variance metric than skin-to-skin time. The early inflection point therefore says more about the speed of technology transfer for an already proficient laparoscopist, and about the choice of CUSUM input variable, than about the de novo learning burden of r-TAPP. The console-versus-skin-to-skin distinction is not trivial: where Kudsi et al. [9] reported both metrics, console time was systematically about 25% shorter than skin-to-skin time and showed lower variance, suggesting that studies relying on console time may locate inflection points earlier than studies using skin-to-skin time, independent of any actual difference in skill acquisition.

At the other end of the range, the 138-case threshold of Kudsi et al. [9] reflects two compounding factors. First, that series — a single surgeon building a robotic programme between 2013 and 2018, before structured proctorship and dedicated robotic teams were widely available — has the longest enrolment period in the dataset. Second, and more importantly, Kudsi et al. used both a standard CUSUM on operative time and a risk-adjusted CUSUM incorporating complications and surgical site events. The 138-case threshold therefore identifies a different conceptual endpoint: not simply when operative efficiency stabilises, but when efficiency and perioperative safety stabilise simultaneously. Read in this light, the Kudsi figure is not best understood as a statistical outlier but as the answer to a more demanding question. Treating it as one valid data point in the dataset — representing a more conservative definition of proficiency — and reporting an additional sensitivity estimate under a homogeneous methodological framework allows both interpretations to coexist.

Once these contextual differences are taken into account, the available evidence is broadly compatible with the operative-time stabilisation point falling somewhere in the range of approximately 25–35 procedures for surgeons with prior minimally invasive experience operating in a contemporary training setting. We frame this range as a hypothesis-generating observation rather than as a benchmark: the underlying evidence is exclusively retrospective, the certainty is very low, and the inflection points being summarised do not all measure the same thing. The figure is broadly consistent with the 20–50 cases reported for laparoscopic TAPP [1, 23] and would, if confirmed prospectively, suggest that the additional learning burden of transitioning to the robot is modest for surgeons already comfortable with the laparoscopic approach. The two surgeons in Solaini et al. (2025) [22] had backgrounds in robotic colorectal surgery rather than laparoscopic TAPP, suggesting that general robotic platform familiarity may also partly compensate for the lack of procedure-specific laparoscopic experience.

The Solaini et al. (2023) [21] series offers the only direct within-study comparison of expert and trainee learning curves in r-TAPP, and the result is striking: the trainee (Surgeon B) reached stabilisation at 20 cases, while the expert (Surgeon A) needed 35 — despite the trainee’s consistently longer operative times. The authors attributed this to structured mentorship in a high-volume robotic centre. We emphasise that this observation derives from a single study with two surgeons; it is consistent with the broader surgical education literature on structured proctorship but cannot be considered definitive evidence that mentorship compresses the r-TAPP learning curve. It does, however, raise the testable hypothesis that the quality of the training environment is a stronger determinant of the inflection point than the trainee’s baseline experience.

Pre-clinical preparation appears to be a common feature of the more efficient learning trajectories in our dataset. Five of seven studies described some combination of simulator training, dry- and wet-lab sessions, cadaver or animal labs, and proctored initial cases [9, 12, 13, 21, 22]; the Mimic dV-Trainer was used in Proietti et al. [13], the trainee in Solaini et al. (2023) [21] completed simulator and > 150 assisted procedures before the first independent r-TAPP, and Celotto et al. [12] documented cadaver and porcine lab work prior to clinical SP-TAPP. While the small number of studies and the absence of standardised reporting preclude a quantitative assessment of the contribution of simulation, the consistency of the pattern is suggestive and aligns with the European Hernia Society and EAES recommendations on structured robotic training pathways [6, 7]. Celotto et al. [12] further illustrate the role of platform skill transfer: their experienced SP-robotic surgeon achieved early stabilisation in just 13 SP-TAPP cases, confirming that skills acquired on related robotic platforms are a powerful accelerant. The secondary inflection point at 113 cases in that series reflects high-level optimisation, not basic stabilisation, and should not be conflated with the primary thresholds reported elsewhere.

The clinical heterogeneity across the included studies is real and identifiable. First, operative time was defined inconsistently: five studies used skin-to-skin time, one console time, one a normalised metric adjusted for bilateral cases. Second, case complexity varied widely: bilateral rates ranged from 0% to 49%, and complex hernia proportions from 6% to 21%. Solaini et al. (2025) [22] formally quantified this in a mixed-effects model: EHS grade 3 hernias added 21.8 min (*p* < 0.001) and recurrent hernias 6.2 min (*p* = 0.042), independent of case number, while BMI was not significantly associated with operative time. Third, absolute operative times differed by up to 85 min in the pre-learning phase (36 min for Choi [20] vs. 120 min for Aghayeva [11]) — a gap that reflects procedure scope and case selection, not proficiency. These observations are the main reason we deliberately avoided producing a pooled operative-time effect size: any single summary figure would obscure these substantive clinical differences. Future studies should report skin-to-skin time, console time, and docking time separately, stratified by laterality and complexity.

From a safety standpoint, the included evidence is reassuring but not conclusive. Conversions to open or laparoscopic surgery were not reported in any of the studies that addressed this outcome, across a combined 763 procedures. Intraoperative events were rare (four across 1361 procedures: one orchiectomy, one Veress-related stomach injury, one serosal tear and one bladder injury), all manageable, and not systematically clustered in early experience. Postoperative complications were predominantly minor (seromas, haematomas, urinary retention), with major events (Clavien–Dindo ≥ III) infrequent. The largest series [9] showed a statistically significant reduction in surgical site events after the stabilisation point; the others were individually underpowered to detect such differences, and the apparent crude reduction in pooled complication rates (14.1% pre vs. 10.0% post) cannot support a causal inference. Importantly, none of the included studies stratified longer-term outcomes — recurrence, chronic groin pain, or return to activity — by learning-curve phase. Inguinal hernia repair is a procedure whose ultimate quality is defined by long-term outcomes, and the reliance on operative time as the primary surrogate is a substantive limitation of the field, not merely of this review.

Taken together, the available data are compatible with a supervised series of approximately 25–35 r-TAPP procedures being a reasonable hypothesis-generating range for surgeons with prior minimally invasive experience entering a structured training programme. We emphasise that this is a hypothesis to be tested prospectively, not a credentialing benchmark. For surgeons without prior TAPP or robotic experience, a longer trajectory should be expected — plausibly 40–60 cases — and formal mentored proctorship with ongoing CUSUM monitoring is advisable. The Kudsi series [9], when read as a more conservative definition of proficiency rather than as an outlier, provides a useful upper-bound reference for what the curve can look like when both efficiency and safety are tracked simultaneously.

We are not aware of any evidence-based credentialing standard specifically for robotic inguinal hernia surgery published by EHS, EAES, or SAGES. The present findings may contribute to the development of such standards, but they should be regarded as preliminary: the evidence base is small, exclusively retrospective, and graded as very low certainty. Any institutional application should be adapted to local context, surgeon background, and available mentorship resources, and should be validated prospectively.

At a practical level, robotic hernia programmes are probably best launched by concentrating early volume in a small number of designated surgeons rather than spreading cases thinly across a department. This approach shortens the learning period and limits the number of patients exposed to the early part of the curve.

### Limitations

This review has several limitations. First, all included studies are retrospective, with the inherent risks of selection bias and inconsistent outcome ascertainment; none of the included series specified the proportion of inguinal hernia repairs performed robotically at the institutional level or the criteria used to allocate cases to a robotic versus laparoscopic approach, which limits the assessment of selection bias. Second, CUSUM was applied heterogeneously: target values, risk-adjustment approaches, and phase boundary criteria differed across studies. Third, individual-level variance estimates for the inflection point are not available, which is why we chose a scoping review framework and used bootstrap confidence intervals on the summary statistics rather than inferential meta-analytic pooling; even descriptive aggregation simplifies methodological differences between studies, a limitation we have made explicit. Fourth, surgeon-level covariates were inconsistently reported — in particular, none of the included studies reported surgeon age or years from completion of training, both of which are plausibly relevant to the speed of robotic skill acquisition. Fifth, anaesthetic protocols (including opioid-free pathways) were not described in any series, precluding any inference on their potential modulation of recovery and discharge timing. Sixth, publication bias is likely: series with rapid learning curves are probably easier to publish, and grey-literature screening can only partly mitigate this. Seventh, some studies counted patients while others counted procedures (bilateral = two), which inflates the inflection point in cohorts with high bilateral rates. Eighth, ‘inflection point’ itself meant different things across studies — end of a learning phase vs. sustained performance standard, with or without safety adjustment — and treating these as a common descriptive marker is a pragmatic approximation. Ninth, all data come from Da Vinci platforms; transferability to alternative robotic systems (Hugo RAS, Versius, Senhance) is unknown. Tenth, no study reported long-term patient-centred outcomes (recurrence, chronic pain, return to activity) stratified by learning phase — the outcomes that ultimately define the quality of an inguinal hernia repair. Eleventh, the analysis is collectively underpowered to detect clinically meaningful differences in safety outcomes between phases, and the absence of statistically significant differences should not be read as evidence of equivalence. Finally, the review addresses adult inguinal hernia exclusively; paediatric anatomical variants (congenital aetiology, peritoneal-vaginal duct persistence) were out of scope.

### Future directions

Prospective multicentre registries with standardised CUSUM methodology and uniform operative time definitions are the most important next step. Studies should pre-specify whether CUSUM is built on operative time alone or on a composite efficiency-plus-safety endpoint, and report both where feasible. Studies comparing the effect of simulation training, proctorship intensity, and prior laparoscopic volume on the learning curve would be particularly valuable. Extending outcome measurement beyond operative time to include phase-stratified recurrence, chronic pain, and return to activity would give a fuller picture of what proficiency really means, and is the key gap exposed by this review. Validation on non-Da Vinci platforms is an emerging priority. Finally, newer technologies — AI-assisted video analysis, automated surgical phase recognition — may eventually complement or even replace CUSUM as tools for learning curve assessment.

## Conclusion

This scoping review offers the first systematic mapping of CUSUM-based learning curve data for r-TAPP. The available evidence is broadly compatible with the operative-time stabilisation point being reached within approximately 25–35 procedures for surgeons with prior minimally invasive experience operating in structured training environments, although this should be regarded as a hypothesis-generating range rather than a benchmark. The wide range of reported thresholds (12–138), the heterogeneity in how stabilisation is defined (with some studies tracking operative efficiency only and others incorporating safety endpoints), and the very low certainty of the evidence all caution against treating this figure as a universal proficiency standard. Prospective multicentre studies that report phase-stratified long-term outcomes — recurrence, chronic pain, and return to activity — are needed before this estimate can inform credentialing or training-pathway design.

## Supplementary Information

Supplementary file 1

## Data Availability

No datasets were generated or analysed during the current study.
