# Supplementary material for: Learning curve of robotic-assisted transabdominal preperitoneal inguinal hernia repair (r-TAPP): a scoping review of CUSUM-based studies
Source: J Robot Surg. 2026 May 26;20(1):535. doi: 10.1007/s11701-026-03482-7 (PMC13201324; doi:10.1007/s11701-026-03482-7)
Supplement: Supplementary file 1 [file 11701_2026_3482_MOESM1_ESM.docx]

**Supplementary Fig. 1s**

**Pubmed (185 results)**

(("Hernia, Inguinal"[Mesh] OR "inguinal hernia"[Title/Abstract])

AND

("Herniorrhaphy"[Mesh] OR "inguinal hernia repair"[Title/Abstract] OR TAPP[Title/Abstract] OR "transabdominal preperitoneal"[Title/Abstract])

AND

(robotic[Title/Abstract] OR robot-assisted[Title/Abstract] OR laparoscopy[Mesh] OR laparoscopic[Title/Abstract])

AND

("Learning Curve"[Mesh] OR "learning curve"[Title/Abstract] OR CUSUM[Title/Abstract] OR "cumulative sum"[Title/Abstract]))

**Embase (98 results)**

('inguinal hernia'/exp OR 'inguinal hernia':ti,ab)

AND

('hernia repair'/exp OR 'herniorrhaphy'/exp OR 'inguinal hernia repair':ti,ab OR tapp:ti,ab OR 'transabdominal preperitoneal':ti,ab)

AND

('robotic surgery'/exp OR robotic:ti,ab OR 'robot assisted':ti,ab)

AND

('learning curve'/exp OR 'learning curve':ti,ab OR cusum:ti,ab OR 'cumulative sum':ti,ab OR 'cumulative sum analysis':ti,ab)

**Scopus (816 results)**

TITLE-ABS-KEY("inguinal hernia" AND ("inguinal hernia repair" OR TAPP OR "transabdominal preperitoneal") AND (robotic OR "robot assisted" OR "robotic surgery") AND ("learning curve" OR CUSUM OR "cumulative sum"))

**Cochrane (6 results)**

"inguinal hernia" AND robotic AND ("learning curve" OR CUSUM OR "cumulative sum")

**Table 1s- Newcastle-Ottawa**

|  | Celotto et al. | Solaini et al. (2025) | Choi et al. | Solaini et al. (2023) | Kudsi et al. | Aghayeva et al. | Proietti et al. |
| --- | --- | --- | --- | --- | --- | --- | --- |
| **Selection** |  |  |  |  |  |  |  |
| Representativeness of the exposed cohort | * | * | * | * | * | * | * |
| Selection of the non exposed cohort | * | * | * | * | * | *  S | * |
| Ascertainement of exposure | * | * | * | * | * | * | * |
| Demonstration that outcome was not present at the beginning of the study | * | * | * | * | * | * | * |
| **Comparability** |  |  |  |  |  |  |  |
| Comparability of the cohorts | * | * | * | * | * | * | * |
| **Outcome** |  |  |  |  |  |  |  |
| Assessment of outcome | * | * | * | * | * | * | * |
| Follow up length | * | * | * | * | * | * | * |
| Follow up adequacy | * | * | * | * | * | * | * |
|  | 8/9 | 8/9 | 8/9 | 8/9 | 8/9 | 8/9 | 8/9 |

**Table 2s. GRADE assessment of the certainty of evidence by outcome.**

| **Outcome** | **No. of studies** | **Study design** | **Risk of bias** | **Inconsistency** | **Indirectness** | **Imprecision** | **Publication bias** | **Certainty of evidence** | **Rationale** |
| --- | --- | --- | --- | --- | --- | --- | --- | --- | --- |
| Number of procedures required to achieve proficiency (CUSUM inflection point) | 7 studies, 8 independent data points | Retrospective observational studies | Serious | Very serious | Serious | Not serious | Suspected | Very low | All included studies were retrospective observational series. The reported inflection point ranged from 12 to 138 procedures, with marked heterogeneity in surgeon experience, institutional context, and CUSUM methodology. In addition, the proficiency threshold was not uniformly defined across studies, and some series reported patients while others reported procedures as the unit of analysis. |
| Operative time reduction after proficiency attainment | 7 studies | Retrospective observational studies | Serious | Very serious | Serious | Not serious | Suspected | Very low | Operative time decreased across studies, and the pooled mean difference was 16.9 minutes, but heterogeneity was very high (I²=96.7%). Important sources of indirectness included different operative time definitions across studies, including skin-to-skin time, console time, and corrected operative time, as well as differences in case complexity and bilateral hernia rates. |
| Perioperative complications | 7 studies | Retrospective observational studies | Serious | Serious | Serious | Serious | Suspected | Very low | Complication data were available across all studies, but follow-up duration and reporting methods were heterogeneous, precluding formal pooling. Although a trend toward fewer complications after proficiency attainment was observed, event rates were low and no individual series showed a statistically significant between-phase difference. |
| Conversion to open or laparoscopic surgery | 5 of 7 studies explicitly reported conversion data | Retrospective observational studies | Serious | Serious | Serious | Very serious | Suspected | Very low | Conversion was explicitly reported in only five studies, with no conversions observed in those series. The certainty is limited by incomplete reporting across studies and by extremely sparse event data, which make the estimate highly imprecise. |
| Length of hospital stay | Reported inconsistently across included studies | Retrospective observational studies | Serious | Serious | Serious | Serious | Suspected | Very low | Length of stay was inconsistently reported and no significant between-phase differences were identified in the studies reporting this outcome. The evidence is limited by observational design, heterogeneous reporting, and imprecision. |

**Abbreviations:** CUSUM, cumulative sum; GRADE, Grading of Recommendations Assessment, Development and Evaluation.

**Explanation of GRADE ratings:** observational studies started at low certainty. The certainty of evidence was downgraded when appropriate for risk of bias, inconsistency, indirectness, imprecision, and suspected publication bias. No outcome was upgraded.
